# Hydroxy-Selenomethionine Improves the Selenium Status and Helps to Maintain Broiler Performances under a High Stocking Density and Heat Stress Conditions through a Better Redox and Immune Response

**DOI:** 10.3390/antiox10101542

**Published:** 2021-09-28

**Authors:** Hua Sun, Ling Zhao, Zi-Jian Xu, Michele De Marco, Mickael Briens, Xiang-Hua Yan, Lv-Hui Sun

**Affiliations:** 1 Hubei Hongshan Laboratory, College of Animal Science and Technology, Huazhong Agricultural University, Wuhan 430070, China; huasun@webmail.hzau.edu.cn (H.S.); zijianxu@webmail.hzau.edu.cn (Z.-J.X.); xhyan@mail.hzau.edu.cn (X.-H.Y.); 2 Adisseo France S.A.S., 10, Place du Général de Gaulle, 92160 Antony, France; michele.demarco@adisseo.com (M.D.M.); Mickael.Briens@adisseo.com (M.B.)

**Keywords:** broilers, hydroxy-selenomethionine, selenoprotein, stocking density, heat stress, health status

## Abstract

This study has determined whether hydroxy-selenomethionine (OH-SeMet) exerts a better protective action on broilers against environmental stress than sodium selenite (SS) or seleno-yeast (SY). Day-old male Cobb 500 broilers (12 cages/diet, 9 broilers/cage) were fed a selenium (Se)-deficient diet (0.047 mg/kg) supplemented with SS, SY or OH-SeMet at 0.3 mg Se/kg under a high stocking density and heat stress condition for six weeks. OH-SeMet improved the FCR and Se concentration in the tissues than SS and SY. SY and OH-SeMet both reduced the serum cortisol, T3, IL-6, IgA, IgM and LPS, more than SS, while only OH-SeMet further increased IL-10 and IgG. SY and OH-SeMet improved the intestinal morphology and increased the T-AOC, TXRND, SELENON and OCCLUDIN activities but decreased CLAUDIN2 in the jejunum than SS, while OH-SeMet further improved these values than SY. SY and OH-SeMet both increased SELENOS and TXNRD2 in the muscles than SS, and OH-SeMet further raised T-AOC, GPX4, SELENOP, SELENOW and TXNRD1, and reduced malondialdehyde and protein carbonyl in the muscles than SS and SY. OH-SeMet showed a better ability to maintain the performance and the redox and immune status of broilers under a high stocking density and heat stress challenge than SS and SY.

## 1. Introduction

Stocking density is an important issue in the broiler industry because of its direct effect on animal welfare and economic profit [1]. A high stocking density is often introduced to increase profitability, as it results in a greater chicken meat yield per fixed raising area [2]. However, a high stocking density, if not well managed, has been reported to decrease broiler productivity by lowering the growth performance and meat quality of broilers as a result of an impaired redox balance, immunity and intestinal health [1,2].

Additionally, heat stress is another serious welfare issue that can causes marked economic losses each year due to a poor performance and poor immunity and health conditions [3,4]. A decreased feed intake, increased excretion and reduced bioavailability of key nutrients during heat stress contribute to reducing the performance and potency of the immune response [3,4,5]. Moreover, high environmental temperatures as well as high relative humidity have been shown to adversely affect the oxidative status of poultry [2,3].

Selenium (Se) is an essential trace element for humans and animals, with key functions in the antioxidant defense and in the immunity and inflammatory response modulation of the body [6]. These metabolic roles of Se have primarily been attributed to its presence in the 21st amino acid: selenocysteine, the core constituent of 25–26 selenoproteins in different animal species [6,7]. Other studies have reported that a dietary supplementation of Se can help to effectively mitigate the negative effects induced by a high stocking density and heat stress in different animal species [8,9,10]. The protective action of Se against a high stocking density and adverse heat stress induced effects, which is related to an adequate Se supplementation of the feed and an optimal Se status of the body, has been shown to be associated with an enhanced antioxidant capacity and immune functions [8,11].

In order to avoid an Se deficiency and to fulfill the requirements and needs of poultry, it is standard practice to supplement feeds with Se in either an inorganic Se form (sodium selenite, SS; blends of SS and soya protein hydrolysates), or organic Se form, the latter of which is widely adopted because of its higher bioavailability [12]. One of the organic forms, Se-yeast (SY, Se-enriched yeast) has been authorized as a feed supplement to provide at least 63% of the total Se as selenomethionine (SeMet) while, more recently, pure chemically synthesized forms of SeMet or hydroxy-selenomethionine (OH-SeMet), have been authorized as feed supplements, the latter providing > 98% of the total Se as OH-SeMet [13,14,15,16].

The main advantage of feeding Se in the form of SeMet over inorganic sources or other organic Se compounds (e.g., selenocysteine, water-soluble selenometabolites etc.) is that SeMet is metabolized as a constituent of the methionine pool, and this leads to a storage depot of Se being created in the body tissues of animals [12]. Increased muscle and tissue reserves of Se can enhance the resistance of livestock to stress and diseases and therefore represent a key strategy to help fight commercially relevant stress [12,17].

To date, the functional benefits of OH-SeMet, compared to both SS and SY, have been described for dairy cows [18,19], swine [20,21], layers [22,23] and broilers [24,25]. Furthermore, it has been demonstrated that OH-SeMet has a unique ability to increase the deposition of Se in the tissues of broilers and to significantly increase the expression and translation of several selenoproteins compared to SS and SY [26]. Since these selenoproteins were upregulated by OH-SeMet, which plays a more important role in the antioxidant defense and immune regulation than SS and SY [7,27], the hypothesis behind the present study was that OH-SeMet could offer extra protection and/or benefits to broiler chickens raised under stressful environmental and management conditions. Therefore, the present experiment has been conducted to (1) determine whether OH-SeMet is able to protect broiler chickens more under such challenging conditions as a high stocking density and heat stress than SS or SY; and (2) explore the underlying mechanisms of such a process.

## 2. Materials and Methods

### 2.1. Chickens, Treaztments, and Sample Collection

Our animal protocol was approved by the Institutional Animal Care and Use Committee of Huazhong Agricultural University, China. The identification code of the project is HZAUCH-2020–0008. Overall, 324 one-day-old Cobb 500 male broiler chickens (12 cages/diet, 9 chicks/cage; cage with an identical floor size of 0.70 m × 0.71 m) were allocated to three experimental groups. As there were approximately 18 growing broilers/m^2^, it is possible to consider that the birds were under high stocking density stress [2]. The birds were allowed free access to water and feeds and were fed a Se-deficient basal diet (BD; Se content: 0.047 mg/kg mg Se/kg; Table S1) supplemented with 0.3 mg Se/kg as SS or SY, or OH-SeMet. The analyzed Se concentrations in the BD supplemented with SS, SY and OH-SeMet were 0.345, 0.351 and 0.338 mg/kg, respectively. The experiment lasted six weeks, and was conducted between 27 July and 7 September 2020 in Wuhan (China) to take advantage of the high temperatures and high relative humidity of that period, which are indicated in Figure S1. The average daily temperature in the chicken house was 33.4 ± 0.7 °C (ranging from 32.7 to 36.0 °C; Figure S1A), and the average relative humidity was 70.9% (ranging from 56.0 to 82.4%; Figure S1B) throughout the experiment.

The health status and mortality of the birds were monitored daily during the trial. The body weight gain (BWG), feed intake (FI) and feed conversion ratio (FCR) were determined every three weeks. Moreover, 12 birds from each treatment group (1 bird/cage) were sacrificed at week six to collect blood, liver, pectoral muscle and small intestinal samples. The samples were washed with ice-cold isotonic saline before being cut with surgical scissors. The samples were divided into aliquots, snap-frozen in liquid nitrogen, and stored at −80 °C until use.

### 2.2. Selenium Concentration and Antioxidant Parameter Analysis

The total Se concentrations in the feeds and in the fresh tissues were measured by means of a hydride generation atomic fluorescence spectrometer (AF-610B; Beijing Rayleigh Analytical Instrument Corporation) [26]. The pectoral muscle and jejunum activities of the total antioxidant capacity (T-AOC) as well as the glutathione peroxidase (GPX) and lipopolysaccharide (LPS), malondialdehyde (MDA) and protein carbonyl (PC) concentrations were measured by means of a colorimetric method, using specific assay kits (H255, A015-1-2, A005, A006-1, A061-1, A003 and A087-1-2) from the Nanjing Jiancheng Bioengineering Institute of China. The thioredoxin reductase (TXNRD) activity was measured by considering the NADPH-dependent reduction of 5,5-dithiobis-(2-nitrobenzoic acid), using a specific assay kit (BW11) from the Suzhou Comin Biotechnology Co., Ltd. of China [26]. The protein concentrations were analyzed through a bicinchoninic acid assay [26].

### 2.3. Serum Biochemical, Immunoglobulin and Histological Analyses

The cortisol, triiodothyronine (T3), thyroxine (T4), (interleukins) IL-6 and 10, tumor necrosis factor α (TNF-α), immunoglobulin (Ig) A, IgG, IgM and endotoxin concentrations in the serum were measured by means of ELISA kits (CSB-E13270C, CSB-E15787C, CSB-E08549Ch, CSB-E12835C, CSB-E11231Ch, CSB-E11232Ch, CSB-EQ027259CH, CSB-E16200C; Cusabio Biotech Co. Ltd. Wuhan, China) according to the manufacturer’s instructions. The small intestine tissue samples were microscopically examined after being fixed in 10% neutral buffered formalin, embedded in paraffin, sectioned at 5 μm, and then stained with hematoxylin and eosin [28]. The diamine oxidase activity in the serum (DAO) was determined by means of a colorimetric method using specific assay kits (A088-1-1; Nanjing Jiancheng Bioengineering Institute of China, Nanjing, China).

### 2.4. Western-Blot Analyses

Western blot analyses of the muscle and jejunum samples were performed as previously described [29]. The primary antibodies used for each gene are presented in Supplemental Table S2. The specificity and reliability of individual antibodies against the specific proteins were validated (Figure S2). The protein concentrations were measured through a bicinchoninic acid assay [26]. The relative density of the protein band was quantified using the Alpha-Imager 2200 system (Alpha Innotech, San Leandro, CA, USA).

### 2.5. Statistical Analysis

Statistical analysis was performed using SPSS (version 13). The obtained data are presented as the mean ± SE. Dietary effects were determined, by means of one-way ANOVA, using a significance level of *p* < 0.05, and the Tukey-Kramer method was used for multiple mean comparisons.

## 3. Results

### 3.1. Growth Performance and Deposition of Selenium

**Figure 1:**
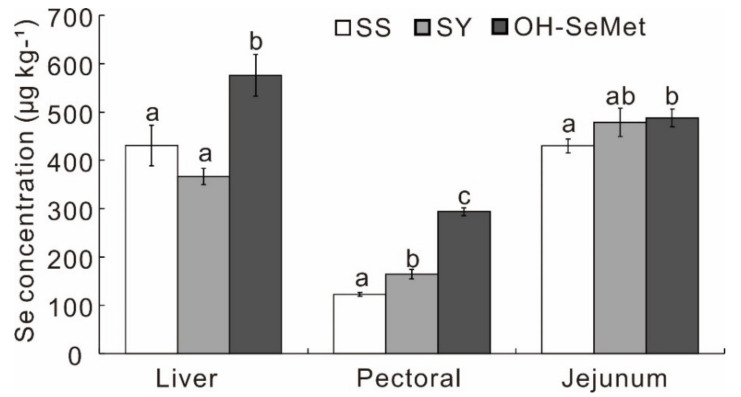
Effect of the three Se forms on the total Se in the fresh liver, pectoral muscle and jejunum samples of the chicks. The values are the means ± SE, *n* = 12. Labeled means within the same plot without a common letter differ, *p* < 0.05. SS, basal diet supplemented with 0.3 mg Se/kg as sodium selenite; SY, basal diet supplemented with 0.3 mg Se/kg as seleno-yeast; OH-SeMet, basal diet supplemented with 0.3 mg Se/kg as hydroxy-selenomethionine.

After 6 weeks of the experimental treatments, the growth performance variables were significantly affected by the different forms of Se (Table 1). OH-SeMet improved (*p* < 0.05) the BWG (+28 g; +4.8%) during weeks 1–3 and FCR (−3 points; −2.2% and −1.8%, respectively) during weeks 1–3 and 1–6 more than SS. SY fell somewhere between SS and OH-SeMet for FCR during weeks 1–6. OH-SeMet also significantly increased (*p* < 0.05) the BWG (+29 g; +5.0%) and showed a significant better FCR than the SY group (*p* < 0.05; −3 points; −1.8%) during weeks 1–3. The mortality of the chicks was not affected (*p* ≥ 0.05) by the three forms of Se. Only OH-SeMet enhanced (*p* < 0.05) the Se concentration, by 31.8% in the liver and 13.5% in the jejunum, compared to SS, while both SY and OH-SeMet enhanced (*p* < 0.05) the Se concentration by 34.1–139% in the pectoral muscle (Figure 1). Furthermore, OH-SeMet resulted in 57.0% and 78.3% greater (*p* < 0.05) Se concentrations in the liver and pectoral muscle, respectively, than SY.

### 3.2. Serum Biochemistry and Small Intestinal Histology

**Figure 2:**
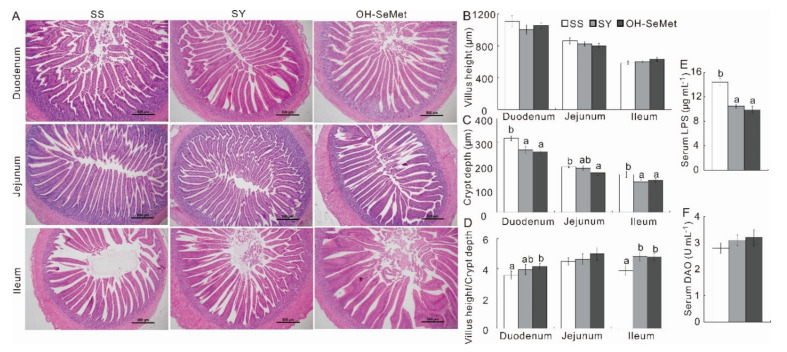
Effect of the three forms of Se on the histology of the intestine (**A**), villus height (**B**), crypt depth (**C**), villus height/ crypt depth ratio (**D**), Serum LPS (**E**) and Serum DAO (**F**) of the chicks. The values are the means ± SE, *n* = 12. Labeled means within the same plot without a common letter differ, *p* < 0.05. SS, basal diet supplemented with 0.3 mg Se/kg as sodium selenite; SY, basal diet supplemented with 0.3 mg Se/kg as seleno-yeast; OH-SeMet, basal diet supplemented with 0.3 mg Se/kg as hydroxy-selenomethionine.

The effects of the different Se sources on the hormones, cytokines and immunoglobulin in the serum are presented in Table 2. SY and OH-SeMet both reduced (*p* < 0.05) the cortisol, T3 and IL-6 in the serum more than SS. Interestingly, only OH-SeMet increased (*p* < 0.05) IL-10, by 34.8–42.5%, in the serum more than SS and SY. OH-SeMet significantly increased the IgG serum concentration (*p* < 0.05) more than both SS and SY, which showed equivalent values (*p* ≥ 0.05). SY and OH-SeMet both decreased the serum levels of IgM (*p* < 0.05) more than SS, while the IgA serum concentrations were significantly reduced from SS to SY to OH-SeMet (*p* < 0.05). No significant differences (*p* ≥ 0.05) were found for the small intestine histopathological changes between the 3 groups (Figure 2A). SY and OH-SeMet both decreased (*p* < 0.05) the crypt depth in the duodenum and ileum, by 15.5–19.3%, more than SS, while only OH-SeMet decreased (*p* < 0.05) the crypt depth in the jejunum, by 13.4% (Figure 2C). SY and OH-SeMet both increased (*p* < 0.05) the villus height/crypt depth ratio in the ileum, by 23.0–24.0%, while only OH-SeMet increased (*p* < 0.05) the villus height/crypt depth ratio, by 17.3%, in the duodenum, more than SS; SY led to values somewhere between SS and OH-SeMet (Figure 2D). Moreover, both SY and OH-SeMet reduced (*p* < 0.05) LPS in the serum, by 27.1–31.3%, more than SS, (Figure 2E). No difference in the DAO in the serum was detected between treatments (*p* ≥ 0.05) (Figure 2F).

### 3.3. Redox Status and Production of Selenoprotein and Tight Junction-Related Proteins in the Jejunum

**Figure 3:**
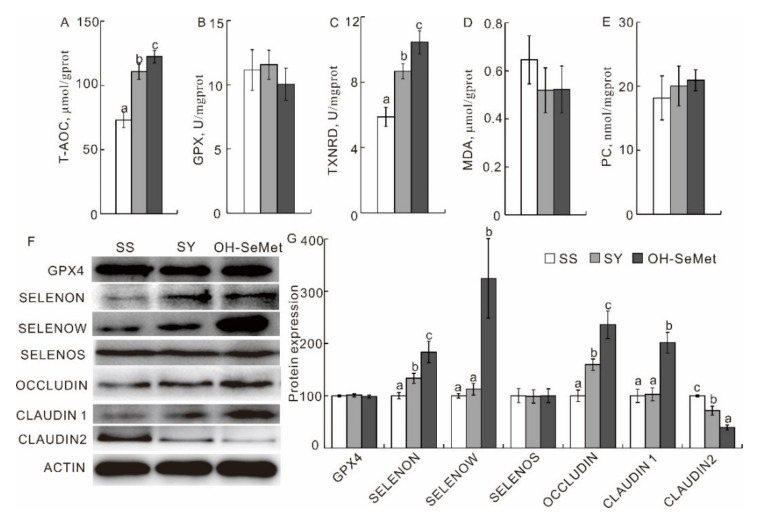
Effect of the three forms of Se on the T-AOC (**A**), GPX (**B**), TXNRD (**C**), MDA concentration (**D**), PC concentration (**E**), and protein production of GPX4, SELENON, SELENOW, SELENOS, OCCLUDIN, CLAUDIN1 and CLAUDIN2 (**F**,**G**) activities in the jejunum of broilers. The values are the means ± SE, *n* = 12 for the redox status, *n* = 3 for the western blot. Labeled means within the same plot without a common letter differ, *p* < 0.05. ACTIN, β-actin; GPX, GPX4, glutathione peroxidase, glutathione peroxidase 4; MDA, malondialdehyde; PC, protein carbonyl; SELENON, S, W, selenoprotein N, S, W; T-AOC, total antioxidant capacity; TXNRD, thioredoxin reductase; SS, basal diet supplemented with 0.3 mg Se/kg as sodium selenite; SY, basal diet supplemented with 0.3 mg Se/kg as seleno-yeast; OH-SeMet, basal diet supplemented with 0.3 mg Se/kg as hydroxy-selenomethionine.

The effects of the different forms of Se on the jejunum redox status are shown in Figure 3A–E. SY and OH-SeMet both enhanced (*p* < 0.05) the T-AOC and TXNRD activity in the jejunum, by 52.1–79.5% and 47.3–82.0%, respectively, more than SS. Furthermore, OH-SeMet resulted in a significantly higher (*p* < 0.05) activity of T-AOC and TXNRD in the jejunum (+18.0% and +23.6%, respectively) than SY. SY and OH-SeMet both upregulated (*p* < 0.05) the protein abundance of SELENON and OCCLUDIN and downregulated (*p* < 0.05) the protein abundance of CLAUDIN2 more than SS. Moreover, between the two tested organic Se sources, the OH-SeMet-fed group showed a significantly higher protein abundance of SELENON, SELENOW, CLAUDIN 1 and OCCLUDIN and a significantly lower protein abundance of CLAUDIN2 than the SY-fed group (*p* < 0.05; Figure 3F,G).

### 3.4. Redox Status and Production of Selenoproteins in the Pectoral Muscle

**Figure 4:**
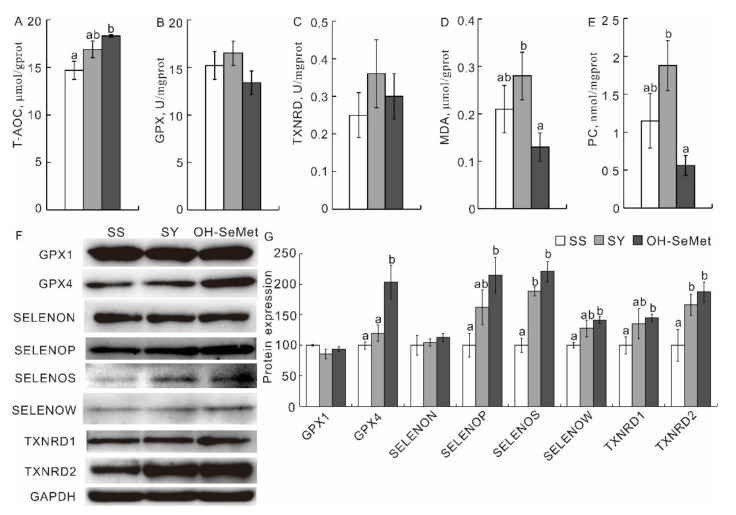
Effect of the three forms of Se on the T-AOC (**A**), GPX (**B**), TXNRD (**C**), MDA concentration (**D**), PC concentration (**E**), and protein production of GPX1, GPX4, SELENON, SELENOP, SELENOS, SELENOW, TXNRD1 and TXNRD2 (**F**,**G**) activities in the pectoral muscle of the broilers. The values are the means ± SE, *n* = 12 for the redox status, *n* = 3 for the western blot. Labeled means within the same plot without a common letter differ, *p* < 0.05. GAPDH, glyceraldehyde-3-phosphate dehydrogenase; GPX, GPX1 and 4, glutathione peroxidase, 1 and 4; MDA, malondialdehyde; PC, protein carbonyl; SELENON, P, S, W, selenoprotein N, P, S, W; T-AOC, total antioxidant capacity; TXNRD, TXNRD1 and 2, thioredoxin reductase, thioredoxin reductase 1 and 2; SS, basal diet supplemented with 0.3 mg Se/kg as sodium selenite; SY, basal diet supplemented with 0.3 mg Se/kg as seleno-yeast; OH-SeMet, basal diet supplemented with 0.3 mg Se/kg as hydroxy-selenomethionine.

The effects of different Se forms on the redox status of the pectoral muscle are presented in Figure 4A–E. Only OH-SeMet increased (*p* < 0.05) the T-AOC, by 24.5%, more than SS, while SY was somewhere between SS and OH-SeMet. OH-SeMet significantly reduced (*p* < 0.05) the muscle MDA and PC, compared to SY, with SS showing intermediate values between the two organic Se sources. SY and OH-SeMet both upregulated (*p* < 0.05) the protein abundance of SELENOS and TXNRD2, compared to SS, but at the same time only OH-SeMet upregulated (*p* < 0.05) SELENOP, SELENOW and TXRND1, while SY was somewhere between SS and OH-SeMet (*p* > 0.05). Lastly, OH-SeMet showed a higher abundance of GPX4 (*p* < 0.05; Figure 4F,G) than SS or SY.

## 4. Discussion

The results of this study have demonstrated that an Se supplementation, in the form of OH-SeMet, is better able to maintain the growth performances of broiler chickens raised under heat stress conditions and a high stocking density than the SS or SY forms. The poor performances of the broilers, induced by heat stress, are mainly related to a depressed feed intake as well as various consequences on the physiology and metabolism of the birds, such as increased oxidative stress [30]. The high temperature and relative humidity induced in the region and by the season where the study took place can explain the globally low growth performances recorded in the present experiment, compared to the standard of the genetic [31]. A high stocking density is also known to further degrade the performance of birds/animals when combined with heat stress [2]. Selenium has often been cited [9,10] in attempts to identify nutritional solutions against heat stress, even though some reviews have not systematically reported any effect of Se supplementation on maintaining performance [9]. However, a recently conducted meta-analysis has confirmed that an optimal Se supplementation helps to maintain the performance of broilers under heat stress and contributes to a better FCR in particular [32]. The present study has gone further by comparing a mineral Se source with two organic Se sources and has shown OH-SeMet to be better able than SS and SY at maintaining an optimal FCR and BWG during the starter and the overall periods. These results confirm previous observations whereby OH-SeMet maintained a better FCR in broilers [33] than SS or SY, under heat stress conditions, as well as in egg production and FCR in layers [34] and in the milk yield of dairy cows [35]. These findings confirm that the two organic Se sources OH-SeMet and SY are not equivalent and that, more than the total Se of those sources, it is their SeMet proportion that mainly drives their efficacy [25].

The hierarchy of efficacy of these Se sources was also confirmed by the Se concentrations in the muscles, as has already been described for cases in which pure organic Se forms of OH-SeMet have a higher bioavailability than SY or SS [12,25]. This efficacy supports the concept that organic Se forms, thanks to the storage of SeMet in the tissues, can constitute a reservoir of Se that becomes available in challenging situations (eg. heat stress) and confirms the superiority of OH-SeMet over SY for a higher storage depot of Se [25,26]. Such a reservoir of Se in heat stress conditions, which may result in increased oxidative stress and a lower availability of feed nutrients due to a limitation of the feed intake, could explain the ability of OH-SeMet to help animals maintain or increase the synthesis of selenoproteins and, therefore, to better support endogenous redox systems, which help to maintain the performance, growth, feed efficiency and overall health and welfare status of animals [17].

The effect of the different Se sources on the oxidative stress response also revealed a greater ability of organic Se sources to limit cortisol and the active thyroid hormone (T3), which indicates a better ability of the animal to adapt and cope with the stress induced by a high stocking density and hot environments [30,36,37]. Hormones, particularly those generated by the adrenal and thyroid glands, play a pivotal role in the thermoregulation and metabolic adjustments of animals [36]. Cortisol and corticosterone are released from the adrenal cortex in response to different stresses, and they affect different immune functions, the inflammatory response and the endocrine systems [30,36,37]. Cortisol, the most important glucocorticoid, has been found to be immunosuppressive, thus inhibiting the production and actions of antibodies, the lymphocyte function and the leucocyte population [38]. A few studies have already reported the beneficial effect of Se on the plasma cortisol level of heat-stressed broilers [39]. However, to the best of our knowledge, this is the first study that has shown a significant difference between inorganic and organic forms of Se. Moreover, Se is directly involved the thyroid hormone metabolism, since the function of three selenoproteins, the iodothyronine deiodinases, is to catalyze the inactive hormone T4 into the active hormone T3 [7,27]. Therefore, Se, as a result of the indirect regulation of thyroid hormones, participates in the basal metabolic rate, protein synthesis, and the fat, carbohydrate, protein and vitamin metabolisms. Thus, maintaining an optimal Se status in the body helps to effectively regulate the synthesis of thyroid hormones and restores body homeostasis, particularly under specific physiological conditions and under pathological or environmental stimuli, when the synthesis and regulation of thyroid hormones may be impaired [7,10]. As the release of cortisol into the circulatory system is a classical response to stress [39,40], and thyroid hormones (T3) are the principal determinants of the metabolic rate and heat production of a body [30,36], our observations seem to indicate that organic forms of Se, and OH-SeMet, in particular, could help animals to better adapt to such conditions.

Oxidative stress is one of the major mechanisms of heat stress-mediated tissue damage [30], and the pectoralis major muscle, a key economic product of broilers, is thus susceptible to environmental stressors [2]. The present study has shown that, of the two tested organic Se sources, only OH-SeMet exerted a better ability to protect the muscle against environmental stressors than SS, as pointed out by the enhanced T-AOC and reduced MDA and PC. Bakhshalinejad et al., (2012) also reported an improved T-AOC in broilers with organic Se forms (SY or SeMet), compared to SS [41]; in the same study, lower MDA levels were observed in various tissues after the administration of SeMet than when SY was administered, and thus our observations on OH-SeMet and SY corroborate their observations.

In the present study, among the assayed selenogenomes, SELENOS and TXNRD2 were upgraded more in the pectoral muscle by SY and OH-SeMet than by SS. Moreover, OH-SeMet upregulated GPX4, SELENOP, SELENOW and TXNRD1 more than SY, thus confirming the results of a previous study [26]; these selenoproteins play important roles in the response to oxidative stress [7,26,27]. Interestingly, the study by Zhao et al. (2017) was conducted under standard environmental conditions and did not reveal any feed conversion differences, which could indicate that an optimal Se status and supply are particularly important under challenging environmental and management conditions that induce oxidative stress, as in the present study where FCR and BWG were improved overall by OH-SeMet.

Gut integrity is also described as a major issue in the case of heat stress, and can directly affect the performances of animals by impairing the morphology, permeability and functioning of the intestine [1,2]. In fact, animals suffering from stress are more susceptible to inflammation and severe infections, because stress hormones increase mucosa permeability, and animals may thus carry more pathogens in the gut and associated lymphoid tissue [42]. The blood of animals is redistributed away from inner organs/tissues to peripheral tissue as an adaptation to high environmental temperatures in order to maximize the dissipation of radiant heat. In this regard, it has been demonstrated that heat stress reduces the intestinal blood flow and induces damage of the intestinal mucosa as a result of oxidative stress, causing a disruption of the intestinal barrier and the risk of endotoxemia [1,2,43]. In the present study, OH-SeMet exerted a better ability to protect the jejunum against environmental stressors than both SY and SS as pointed out by the enhanced T-AOC, which confirmed the observations made in the present study in the muscles and those observed in a previous study in various tissues [41].

The broilers fed the two organic forms of Se, and in particular those fed OH-SeMet, showed a better intestinal morphology and integrity, as indicated by a reduced crypt depth but increased villus height/crypt depth ratio in the duodenum, jejunum and/or ileum, along with an improved intestinal barrier (reduced endotoxin in the serum) than those fed SS. The increased activity of the thioredoxin system, as suggested by the increased TXNRD activity in the OH-SeMet-fed group, compared to SS and SY, also suggests a better activation of the antioxidant response capabilities to fight oxidative stress and maintain gut integrity. This was also confirmed by a thigh junction assessment with OCCLUDIN and CLAUDIN1, which have a tightness function, while CLAUDIN2 plays the role of a pore forming claudin [44]. In fact, the results of the present study have shown that OH-SeMet and SY both upregulated OCCLUDIN and CLAUDIN1 and downregulated CLAUDIN2 in the jejunum more than SS. However, of the two organic Se sources, only OH-SeMet was able to further significantly upregulate OCCLUDIN and showed the lowest CLAUDIN 2 level. These results seem to corroborate the idea that OH-SeMet could induce a better intestinal integrity than SS or SY. Tang et al., (2019) also reported a better protective effect of pure SeMet than SS in a porcine epithelial cell model after the induction of heat stress [45]. Moreover, the increased quantification of SELENON and SELENOW in the jejunum, with specific functions related to oxidative stress, would also seem to indicate a better capability of OH-SeMet to protect the gut from oxidative damage during challenges [7,27].

In addition to the quantification of the tight junction protein and intestinal morphology, increased gut permeability can be recognized by the leakage of such endotoxins as LPS into the blood [43,46]. Our results indicate a lower plasma LPS concentration for OH-SeMet and SY than for SS, which would seem to confirm an improvement of the intestinal integrity. IgA, the most abundant immunoglobulin at the mucosal level, was also present in smaller amounts at the serum level in the OH-SeMet-fed group, which would seem to confirm a higher gut integrity [47]. However, it could indicate a greater passage of pathogens through the intestinal barrier, for SY and in particular for SS, which triggers a more innate and acquired immune system. In parallel, serum IgG was more abundant in the OH-SeMet-fed group than in the SY and SS groups. A general high sensitivity to oxidative stress of the immune cells may have reduced the production of IgG in the latter two groups [48]. However, the higher level of IgG observed for OH-SeMet may demonstrate that the animals were able to maintain a better production of IgG, even under such an oxidative stress situation. On the other hand, the IgM level was lower for the two organic forms of Se, and this may be attributed to an immunoglobulin class switching recombination mechanism, as the B cell switches the production of immunoglobulin from IgM to IgG [49]. This mechanism helps to increase the affinity of the immunoglobulin for antigen [50]. Moreover, IgG is known to be the most abundant immunoglobulin produced by animals, as confirmed by the relative quantities of the three Ig measured in the serum in the present study [51]. Several studies that compared an Se deficient diet with a Se supplemented diet have found that Se significantly improved the immunoglobulin production and humoral immunity [52,53]. The results of this study corroborate those of Kovalenko et al., (2008) who reported that the dietary supplementation of organic Se improved immunoglobulin production in chickens vaccinated against Newcastle disease more than SS [54]. Overall, these results show the beneficial effect of the pure organic form of Se-OH-SeMeton the humoral immune response, which could decrease the susceptibility of animals to infectious diseases, and confirm that a fine-tuned Se supplementation should be part of the solutions for an optimal immune response under heat stress conditions [55].

The cytokine measurements in the present study tended to confirm that organic Se forms, and in particular OH-SeMet, are better able to promote an anti-inflammatory response and consequently decrease inflammation. Indeed, IL-10 is a potent anti-inflammatory cytokine which inhibits the production of primary pro-inflammatory cytokines such as IL-6, and attenuates cell-mediated immune responses [56]. Interestingly, SY and OH-SeMet both reduced the synthesis of pro-inflammatory cytokine IL-6 more than SS; however, only OH-SeMet increased the level of anti-inflammatory cytokine IL-10 in the serum more than SS or SY. These findings reveal that OH-SeMet exerts a better capacity to cope with and adapt to ambient stress in terms of inflammatory response modulation. The present results seem to corroborate those of Li et al., (2020) who found that IL-10 levels were significantly increased 2 h post-LPS simulation in piglets from sows that had received OH-SeMet, compared to those from sows fed SS [21]. Other authors have confirmed that an optimal Se and selenoprotein status, under conditions in which gut inflammation is induced, have been shown to participate in the modulation of the inflammatory response through modulation of NF-kB and the PPARy transcription factor [57].

No GPX activity differences were observed between the tested Se sources in either the muscle or jejunum, which confirms that a drop in GPX activity is an indicator of Se deficiency [58], and that SS would be sufficient to reach a plateau of GPX activity for those tissues [58]. The GPX selenoprotein family are also known to have a lower priority of expression than other selenoproteins [59]. However, other selenoproteins may need a greater availability of Se to ensure expression, activity and functioning, particularly under high oxidative stress conditions, as shown by the greater increase in TXNRD activity for OH-SeMet in the present trial. Other important selenoproteins in the muscles and jejunum are better expressed with SY and even more so with OH-SeMet, as shown in this study and in previous experiments [26]. SELENOW was more abundant in the OH-SeMet group in the muscles and even more so in the jejunum. The precise function of SELENOW is still not clear, but it is known to be related to redox functions, and its high expression in tissues would seem to indicate additional antioxidant properties given by OH-SeMet [7,27].

## 5. Conclusions

Taken together, the results of this study first confirm the hierarchy of the Se bio-efficacy of different Se sources (SS < SY < OH-SeMet) and indicate that, under environmental conditions known to increase oxidative stress in animals, the Se status could be related to differences in the capabilities of the sources to activate various selenoproteins, with functions that are known to play a major role in response to oxidative stress and, consequently, in the inflammatory and immune response of animals. The better capability to fight this oxidative stress is hypothesized to participate in the maintenance of transverse function, as pointed out in this study at the muscle and gut levels, and to finally participate in maintaining the performances of animals under challenging conditions. Finally, the results of this study also reveal that Se sources are not equivalent in promoting an optimal response to oxidative stress, in particular when organic forms of Se with different proportions of SeMet (SY and OH-SeMet) are considered. The higher purity of the active Se compound provided by OH-SeMet explains the different bio-efficacy, performance and health benefits observed in this study from those of SY and SS, and this highlights that it is not enough to only consider the total Se supply, and that the bioavailable Se (SeMet/OH-SeMet) level should also always be considered.

## Figures and Tables

**Table 1:** Effect of the three selenium forms on the growth performances of the chicks ^1^.

	SS	SY	OH-SeMet
d 1 to 21			
BWG, g/bird	579 ± 30 ^a^	578 ± 27 ^a^	607 ± 39 ^b^
FI, g/bird	810 ± 34	807 ± 32	830 ± 36
FCR, g/g	1.40 ± 0.03 ^a^	1.40 ± 0.03 ^a^	1.37 ± 0.03 ^b^
Mortality, %	0 ± 0	0.9 ± 3.2	0 ± 0
d 22 to 42			
BWG, g/bird	958 ± 96	953 ± 142	987 ± 113
FI, g/bird	1855 ± 144	1823 ± 193	1894 ±194
FCR, g/g	1.94 ± 0.07	1.93 ± 0.10	1.92 ± 0.06
Mortality, %	3.7 ± 5.5	3.7 ± 5.5	3.7 ± 7.2
d 1 to 42			
BWG, g/bird	1537 ± 120	1535 ± 160	1594 ± 139
FI, g/bird	2665 ± 174	2633 ± 216	2724 ± 223
FCR, g/g	1.74 ± 0.04 ^a^	1.72 ± 0.06 ^ab^	1.71 ± 0.03 ^b^
Mortality, %	3.7 ± 5.5	4.6 ± 5.5	3.7 ± 7.2

^1^ The values are the means ± SD, *n* = 12. Labeled means in a row without a common letter differ, *p* < 0.05. BWG, body weight gain; FI, feed intake; FCR, feed conversion ratio; SS, basal diet supplemented with 0.3 mg Se/kg as sodium selenite; SY, basal diet supplemented with 0.3 mg Se/kg as seleno-yeast; OH-SeMet, basal diet supplemented with 0.3 mg Se/kg as hydroxy-selenomethionine.

**Table 2:** Effects of the three selenium forms on the biochemistry in the serum of the chicks ^1^.

	SS	SY	OH-SeMet
Hormones			
Cortisol, ng/mL	22.8 ± 2.6 ^a^	20.3 ± 2.5 ^b^	18.9 ± 1.6 ^b^
T3, nmol/L	1.08 ± 0.07 ^a^	0.91 ± 0.13 ^b^	0.84 ± 0.09 ^b^
T4, nmol/L	31.6 ± 2.7	31.7 ± 2.6	31.6 ± 2.6
Cytokines			
IL-6, pg/mL	5.4 ± 0.6 ^a^	4.9 ± 0.4 ^b^	4.3 ± 0.7 ^b^
IL-10, pg/mL	8.7 ± 1.9 ^b^	9.2 ± 1.4 ^b^	12.4 ± 1.5 ^a^
TNF-a, pg/mL	12.9 ± 3.1	11.4 ± 1.4	12.5 ± 2.2
Immunoglobulin			
IgA, ug/mL	53.7 ± 4.4 ^a^	44.5 ± 4.1 ^b^	39.3 ± 4.5 ^c^
IgG, ug/mL	268 ± 55 ^b^	256 ± 29 ^b^	363 ± 28 ^a^
IgM, ug/mL	100.5 ± 10.7 ^a^	85.4 ± 9.7 ^b^	82.0 ± 11.9 ^b^

^1^ The values are the means ± SD, *n* = 12. Labeled means in a row without a common letter differ, *p* < 0.05. SS, basal diet supplemented with 0.3 mg Se/kg as sodium selenite; SY, basal diet supplemented with 0.3 mg Se/kg as seleno-yeast; OH-SeMet, basal diet supplemented with 0.3 mg Se/kg as hydroxy-selenomethionine.

## Data Availability

Data are contained within the article.

## Supplementary Materials

The following are available online at https://www.mdpi.com/article/10.3390/antiox10101542/s1. Table S1: Basal diet formulation and nutritional values; Table S2: Name, type, dilution and source of the primary antibodies; Figure S1: Daily average temperature (A) and humidity (B) of the environment; Figure S2: Validation of the specificity of antibodies against and the correct bands of CLAUDIN1, CLAUDIN2, OCCLUDIN, SELENOW, SELENOP, SELENON, SELENOS, TXNRD1, TXNRD2, GPX1 and GPX4.
