# Supplementary material for: Hydroxy-Selenomethionine Improves the Selenium Status and Helps to Maintain Broiler Performances under a High Stocking Density and Heat Stress Conditions through a Better Redox and Immune Response
Source: Antioxidants (Basel). 2021 Sep 28;10(10):1542. doi: 10.3390/antiox10101542 (PMC8532863; doi:10.3390/antiox10101542)
Supplement: Supplementary file 1 [file antioxidants-10-01542-s001.zip › antioxidants-1388593-supplementary.pdf]

Supplemental Material

Table S1: Basal diet formulation and nutritional values[1].

Ingredients	Content, g/kg
Corn	638
Roasted soybean	300
$CaCO_3$	10.0
$CaHPO_4$	21.0
Salt	3.0
Choline	2.0
Mineral premix[2]	5.0
Vitamin premix[3]	0.5
Amino acid premix[4]	19.6
Total	1000
Nutrient composition (calculated):	
Metabolic energy, MJ/kg	13.4
Crude protein, %	18.0
Lysine, %	1.08
Methionine, %	0.69
Methionine + cysteine, %	0.92
Calcium, %	1.02
Available phosphorus, %	0.47
Selenium, mg/kg	0.047

[1]The analyzed Se concentrations in the BD, SS, SY and OH-SeMet diets were 0.047, 0.345, 0.351, and 0.338 mg/kg, respectively. BD, basal diet; SS, BD supplemented with 0.3 mg Se/kg as sodium selenite; SY, BD supplemented with 0.3 mg Se/kg as seleno-yeast; OH-SeMet, BD supplemented with 0.3 mg Se/kg as hydroxy-selenomethionine.

[2]Trace mineral premix provided per kg diet: $FeSO_4 \bullet 7H_2O$, 379 mg; $CuSO_4 \bullet 5H_2O$, 31.3 mg; $ZnSO_4 \bullet 7H_2O$, 177 mg; $MnSO_4 \bullet 5H_2O$, 154 mg; and KI, 0.5 mg.

[3]Vitamin premix provided per kg diet: retinyl acetate, 1500 IU; cholecalciferol, 200 IU; rac-α-tocopheryl acetate, 50 mg; menadione,5 mg; thiamin, 1.8 mg; riboflavin, 3.6 mg; calcium pantothenate, 10 mg; niacin, 35 mg; pyridoxol, 3.5 mg; *d*-biotin, 0.15 mg; and folacin, 0.55 mg.

[4]Amino acid premix provided per kg diet: *L*-lysine, 4,630 mg; *DL*-methionine, 4,160 mg; *L*-threonine, 2,600 mg; *L*-tryptophan, 355 mg; *L*-isoleucine, 2,010 mg; *L*-valine, 2,435 mg; *L*-phenylalanine, 1,365 mg; and *L*-arginine, 2,570 mg.

Table S2: Name, type, dilution, and source of primary antibodies[1].

Antibody	Isotype	Dilution	Source
GPX1	Rabbit	1:1000	Abcam (Cambridge, MA)
GPX4	Rabbit	1:1000	ABclonal (Wuhan, China)
SELENON	Rabbit	1:1000	ABclonal (Wuhan, China)
SELENOP	Rabbit	1:1000	Abcam (Cambridge, MA)
SELENOS	Rabbit	1:1000	ABclonal (Wuhan, China)
SELENOW	Rabbit	1:1000	Made by our Lab
TXNRD1	Rabbit	1:1000	Abcam (Cambridge, MA)
TXNRD2	Rabbit	1:1000	Abcam (Cambridge, MA)
CLAUDIN1	Rabbit	1:1000	ABclonal (Wuhan, China)
CLAUDIN2	Rabbit	1:1000	ABclonal (Wuhan, China)
OCCLUDIN	Rabbit	1:1000	ABclonal (Wuhan, China)
GAPDH	Rabbit	1:1000	ABclonal (Wuhan, China)
ACTIN	Rabbit	1:10000	ABclonal (Wuhan, China)

[1] ACTIN, β-actin; GAPDH, glyceraldehyde-3-phosphate dehydrogenase; GPX1 and 4, glutathione peroxidase 1 and 4; SELENON, P, S, W, selenoprotein N, P, S, W; TXNRD1 and 2, thioredoxin reductase 1 and 2.

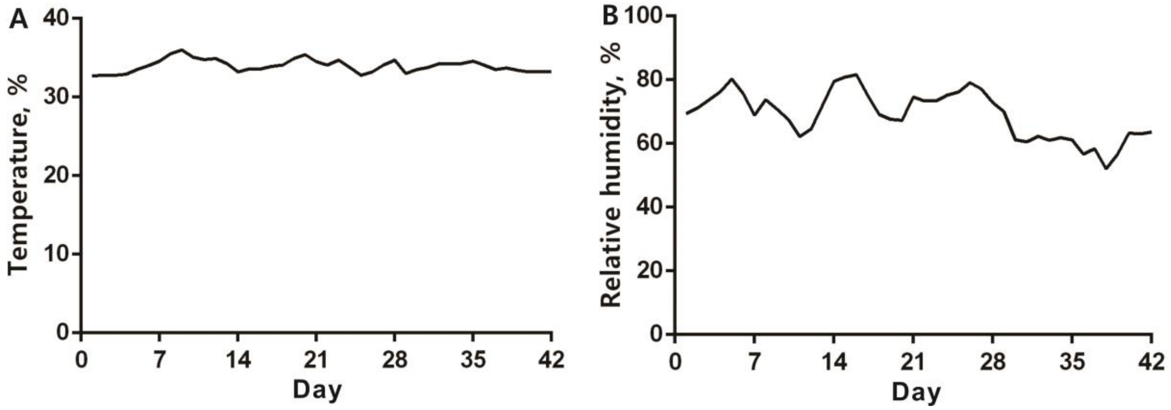

Figure S1: Daily average temperature (A) and humidity (B) of the environment. The temperature and humidity were recorded every hour from 9:00 a.m. to 5:00 p.m., and then the daily average temperature and humidity were calculated by these eight hours.

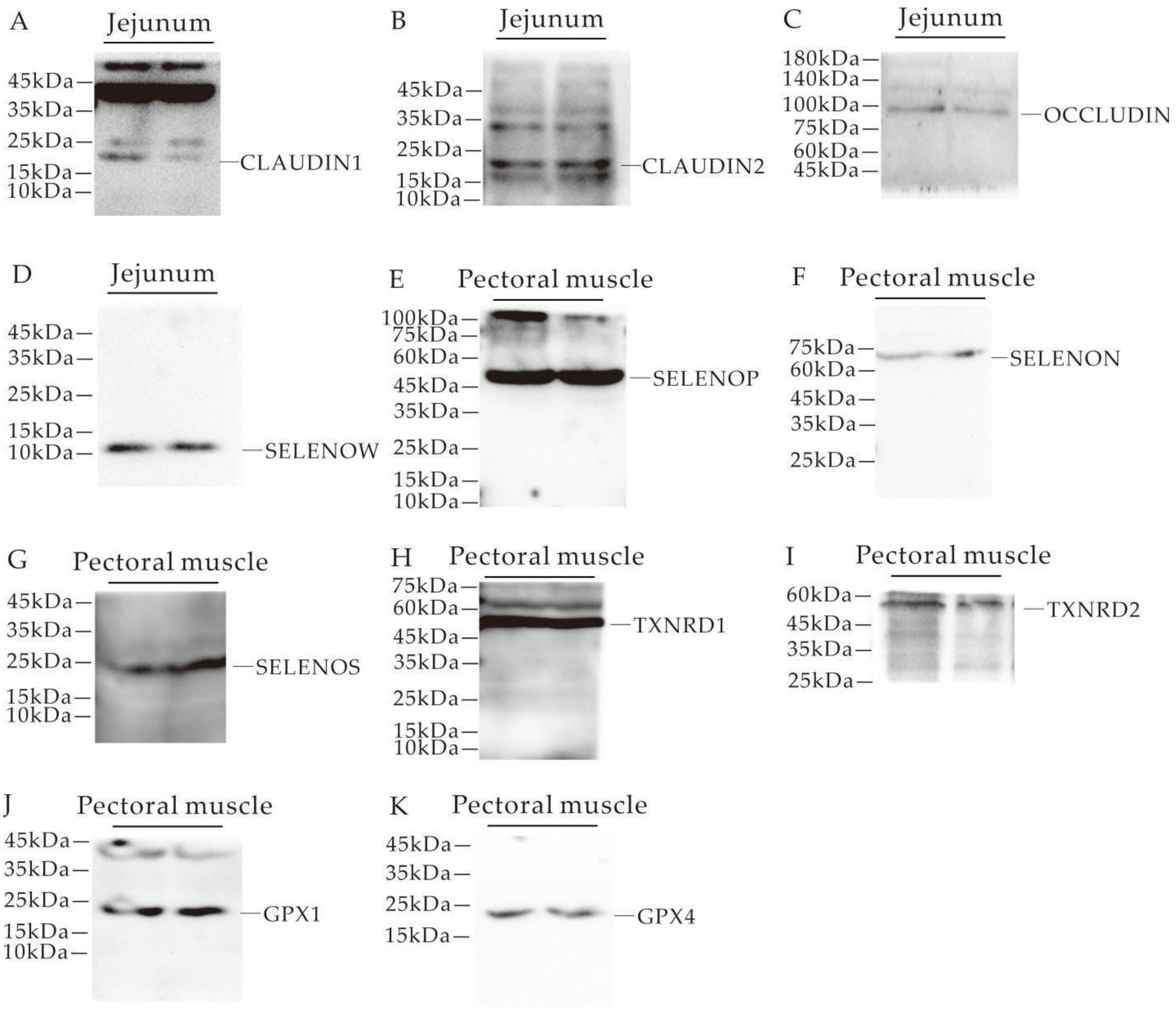

Figure S2: Validation of the specificity of antibodies against and the correct bands of CLAUDIN1, CLAUDIN2, OCCLUDIN, SELENOW, SELENOP, SELENON, SELENOS, TXNRD1, TXNRD2, GPX1 and GPX4, Western blot gel bands of CLAUDIN1 (A), CLAUDIN2 (B), OCCLUDIN (C) and SELENOW (D) were verified with broiler jejunum samples, and SELENOP (E), SELENON (F), SELENOS (G), TXNRD1 (H), TXNRD2 (I), GPX1 (J) and GPX4 (K) were verified with broiler pectoral muscle samples.
